# Chronobiology and Chronotherapy of Osteoporosis

**DOI:** 10.1002/jbm4.10504

**Published:** 2021-05-05

**Authors:** Elizabeth M Winter, Sander Kooijman, Natasha M Appelman‐Dijkstra, Onno C Meijer, Patrick CN Rensen, Maaike Schilperoort

**Affiliations:** ^1^ Department of Medicine, Division of Endocrinology Leiden University Medical Center Leiden The Netherlands; ^2^ Einthoven Laboratory for Experimental Vascular Medicine Leiden The Netherlands; ^3^ Department of Medicine, Center for Bone Quality Leiden University Medical Center Leiden The Netherlands

**Keywords:** BIOLOGICAL CLOCK, BONE REMODELING, CHRONOTHERAPY, CIRCADIAN RHYTHM, FRACTURES, OSTEOPOROSIS

## Abstract

Physiological circadian (ie, 24‐hour) rhythms are critical for bone health. Animal studies have shown that genes involved in the intrinsic molecular clock demonstrate potent circadian expression patterns in bone and that genetic disruption of these clock genes results in a disturbed bone structure and quality. More importantly, circulating markers of bone remodeling show diurnal variation in mice as well as humans, and circadian disruption by, eg, working night shifts is associated with the bone remodeling disorder osteoporosis. In this review, we provide an overview of the current literature on rhythmic bone remodeling and its underlying mechanisms and identify critical knowledge gaps. In addition, we discuss novel (chrono)therapeutic strategies to reduce osteoporosis by utilizing our knowledge on circadian regulation of bone. © 2021 The Authors. *JBMR Plus* published by Wiley Periodicals LLC on behalf of American Society for Bone and Mineral Research.

## 1 Introduction

**Fig 1:**
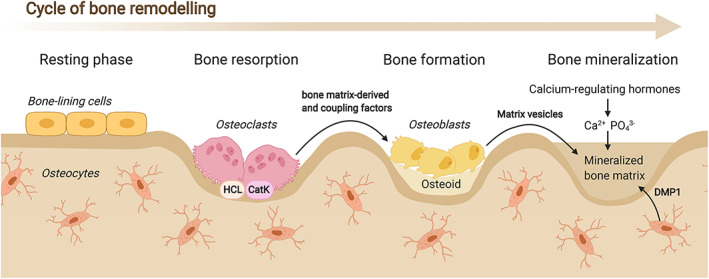
The bone remodeling cycle. See text for explanation.

Healthy bone requires continuous remodeling to maintain its strength.^(^ ^1^ ^)^ This delicate process is dependent on the well‐coordinated activity of osteocytes, osteoclasts, and osteoblasts. After initiation of the bone remodeling cycle by osteocytes in response to mechanical loading or a microfracture, osteoclasts need to be recruited to the bone surface^(^ ^2^ ^)^ to dissolve bone minerals and break down bone matrix. This is achieved by production of hydrochloric acid and lysosomal proteases (eg, cathepsin K),^(^ ^3^ ^)^ which liberates growth factors that are trapped within the bone matrix (eg, bone morphogenetic proteins [BMPs], transforming growth factor beta [TGF‐β], and insulin‐like growth factor 1 [IGF‐1]). Released bone matrix–derived factors promote migration and differentiation of osteoblast precursors.^(^ ^4^, ^5^, ^6^ ^)^ In addition, osteoclasts can also directly interact with osteoblasts via recently identified “osteoclast‐derived coupling factors,” which include cell surface regulatory proteins and secreted factors.^(^ ^7^ ^)^ At the sites of bone resorption, osteoblasts produce new bone matrix predominantly composed of type I collagen. This newly formed osteoid is progressively mineralized by deposition of calcium (Ca^2+^) and phosphate (PO_4_^3−^) in the form of hydroxyapatite crystals to increase mechanical strength of bone.^(^ ^8^, ^9^ ^)^ The mineralization process is facilitated by matrix vesicles produced from osteoblasts^(^ ^10^ ^)^ and osteocyte‐derived proteins such as dentin matrix protein 1 (DMP1),^(^ ^11^ ^)^ after which the bone remodeling cycle is concluded. During this process, some osteoblasts become trapped in the calcified matrix and differentiate into osteocytes, while others turn into bone lining cells (Fig. 1).

Osteoporosis develops if bone resorption exceeds bone formation, leading to bone fragility and increased fracture risk, which can result from either overactivity of osteoclasts^(^ ^12^ ^)^ or hampered osteoblast activity or function.^(^ ^13^ ^)^ The homeostatic activity of bone resorption and bone formation is regulated through the actions of various systemic hormones of which sex hormones are of particular importance. Although in men testosterone is the dominant circulating sex steroid hormone, estrogen is also formed in men from testosterone via aromatization. For women, estrogen is the dominant hormone, but they also have low levels of the androgens that are produced by the adrenal glands.^(^ ^14^, ^15^ ^)^ Androgens affect bone directly by preventing osteoblasts to undergo apoptosis.^(^ ^14^ ^)^ (Androgen‐derived) estrogens inhibit bone resorption by stimulating apoptosis of osteoclasts,^(^ ^16^ ^)^ thus explaining the excessive bone resorption and increased fracture risk in postmenopausal women. Although androgens beneficially affect bone, the action of estrogen is thought to be stronger.^(^ ^14^ ^)^ This is also demonstrated by the fact that blunted estrogen signaling due to mutations in the estrogen receptor gene^(^ ^17^ ^)^ or aromatase deficiency^(^ ^18^, ^19^, ^20^ ^)^ is associated with a lower bone mass in men.

In addition to sex hormones, the whole‐body calcium regulators parathyroid hormone (PTH) and vitamin D are also important for bone health. PTH is produced by the parathyroid gland in response to low serum calcium levels and stimulates the expression of receptor activator of nuclear factor κΒ ligand (RANKL) by osteoblasts.^(^ ^21^ ^)^ RANKL binds to receptor activator of nuclear factor κB (RANK) on osteoclasts, thereby promoting osteoclast proliferation and differentiation. Thus, by stimulating RANK‐RANKL signaling, PTH enhances bone resorption and thereby the release of calcium and phosphate from bone.^(^ ^22^ ^)^ PTH further increases serum calcium levels by promoting reabsorption of urinary calcium in the kidney, and indirectly, by stimulating intestinal calcium resorption. The latter is the result of 1‐alpha‐hydroxylase activity, induced by PTH. This enzyme converts 25‐hydroxycholecalciferol into 1,25‐dihydroxycholecalciferol, ie, the active form of vitamin D, which stimulates calcium and phosphate absorption from the gut.^(^ ^23^ ^)^ The ensuing, immediate rise in serum calcium exerts negative feedback on PTH secretion to ensure that calcium levels are maintained within a narrow range. In addition, high serum calcium levels promote calcitonin production by the thyroid gland. Calcitonin counteracts the effects of PTH by inhibiting bone resorption and increasing renal calcium excretion, although its physiological importance in humans is debated.^(^ ^24^ ^)^

Nutritional status and lifestyle factors fine‐tune the hormonal feedback loops and are therefore also important determinants of bone health.^(^ ^25^ ^)^ Malnutrition, smoking, and excessive alcohol consumption have a detrimental impact on bone, while physical activity promotes bone remodeling. Predominantly during high‐impact and weight‐bearing exercises, mechanical forces are exerted on the bone through ground reaction forces and by the contractile activity of muscles. These forces are sensed by an intricate network of osteocytes, which subsequently respond by shifting the balance in bone remodeling toward bone formation, thereby increasing bone strength.^(^ ^26^ ^)^

**Fig 2:**
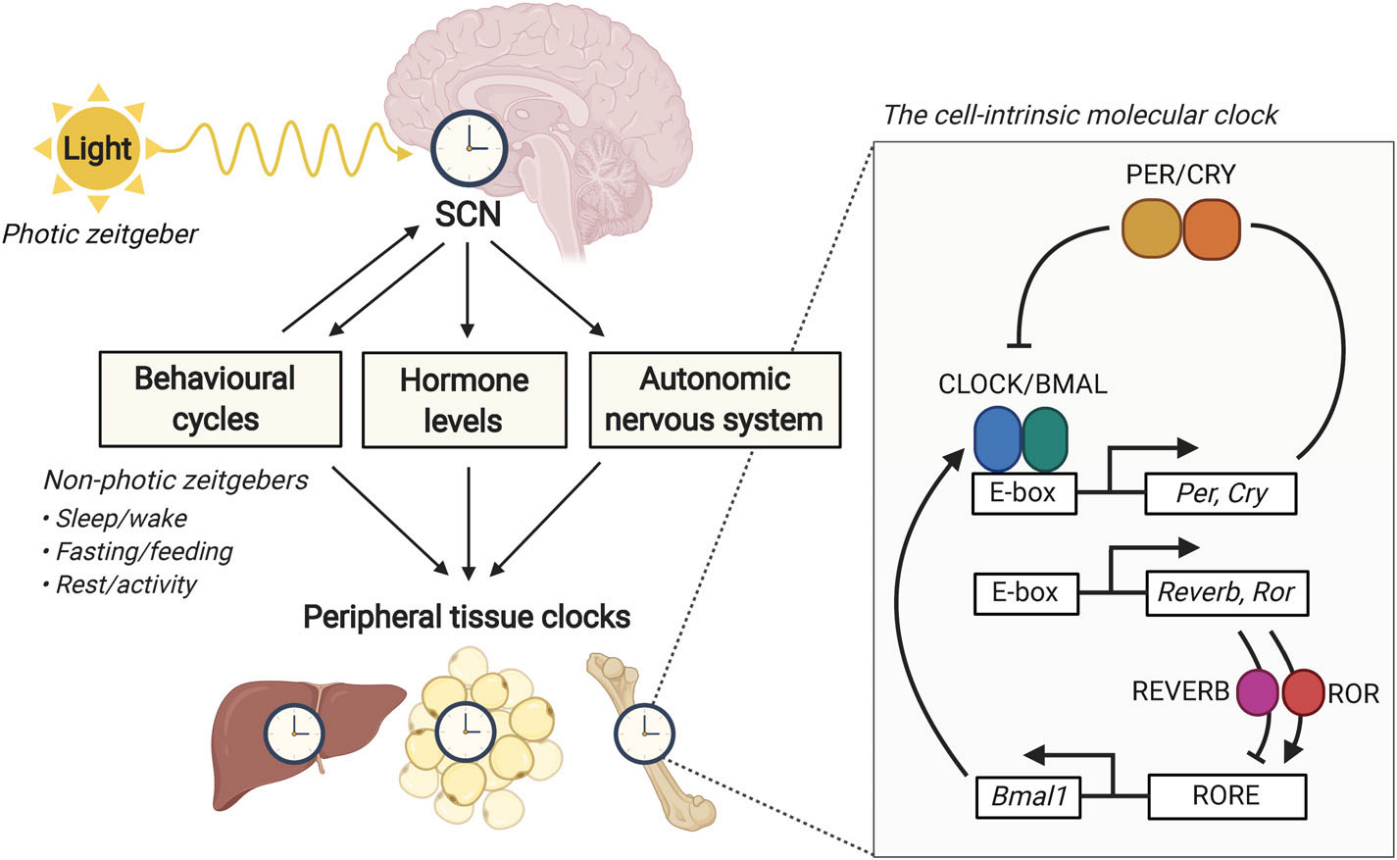
Regulation of the molecular clock in peripheral tissues by the suprachiasmatic nucleus and zeitgebers. The suprachiasmatic nucleus (SCN) receives input from different environmental factors called “zeitgebers” (German for “time‐givers”). Behavioral signals such as sleep/wake, fasting/feeding, and rest/activity can feed back to the SCN and thereby act as “non‐photic zeitgebers.” By receiving input from both photic and non‐photic zeitgebers, the SCN can synchronize robust and coherent circadian rhythms throughout the body. Circadian rhythms within peripheral tissues are maintained by a cell‐intrinsic molecular clock, consisting of clock proteins and clock genes.

It is becoming increasingly clear that bone remodeling is under strict control of the biological clock and that disruption of circadian (ie, 24‐hour) rhythms by night shift work is associated with osteoporosis and fractures.^(^ ^27^, ^28^ ^)^ The circadian timing system orchestrates daily rhythms in physiological processes through a small brain region in the hypothalamus, named the suprachiasmatic nucleus (SCN). The SCN connects the inner workings of the body to the outside environment, by receiving photic (ie, light) and nonphotic input, and orchestrates coherent circadian rhythms in peripheral tissues including bone.^(^ ^29^ ^)^ Transmission of external timing signals is mediated through regulation of autonomic nervous system activity, behavioral cycles (eg, sleep/wake, fasting/feeding, rest/activity), and circulating hormone levels, of which glucocorticoid (GC) hormones are especially important.^(^ ^30^ ^)^ Behavior can also affect circadian clocks in peripheral tissues including bone in an SCN‐independent manner, as described for time‐restricted feeding^(^ ^31^, ^32^, ^33^ ^)^ and scheduled exercise.^(^ ^34^ ^)^ Through these SCN‐dependent and ‐independent mechanisms, illustrated in Fig. 2, body rhythms are synchronized to the external 24‐hour light/dark cycle.

Circadian rhythms within peripheral tissues are maintained through a cell‐autonomous molecular clock (Fig. 2), generated by two interlocking transcriptional/translational feedback loops (TTFL).^(^ ^35^ ^)^ The core TTFL consists of two activator proteins (CLOCK and BMAL1) and two repressor proteins (PER and CRY). The activator proteins CLOCK and BMAL1 heterodimerize and bind to a DNA cis‐element E‐box to initiate transcription of the repressor genes *PER* and *CRY*. PER and CRY proteins subsequently heterodimerize and translocate to the nucleus to inhibit the CLOCK and BMAL1 complex, thereby inhibiting their own transcription, which lasts until they are degraded after 24 hours. This thus results in a self‐sustaining oscillation of core clock genes. The oscillations are fine‐tuned via the induction of clock proteins REV‐ERBα/β and RORα/β by CLOCK/BMAL1 heterodimers, which inhibit and activate *BMAL1* transcription through retinoic acid–related orphan receptor response element (RORE) binding, respectively (Fig. 2). Together, these interlocking feedback loops produce robust 24‐hour rhythms in the expression of genes and proteins comprising the molecular clock (collectively named “clock genes” and “clock proteins”).^(^ ^36^ ^)^ Clock proteins not only regulate the expression of (other) clock genes but also can initiate transcription of tissue‐specific target genes in a circadian oscillating pattern.^(^ ^37^ ^)^ As a result, many important tissue‐specific genes and proteins demonstrate a circadian rhythm.^(^ ^38^, ^39^ ^)^

## 2 Chronobiology of Osteoporosis

We and others have demonstrated that the clock genes *Bmal1*, *Clock*, *Per1*, *Per2*, *Cry1*, and *Reverba* all exhibit diurnal expression patterns in murine calvaria and long bones,^(^ ^40^, ^41^, ^42^ ^)^ indicating that circadian rhythms within bone are indeed maintained through the cell‐autonomous molecular clock. Rhythmic clock gene expression has been detected in cultured osteoclasts^(^ ^42^, ^43^ ^)^ as well as osteoblasts,^(^ ^44^, ^45^, ^46^ ^)^ but it is not yet known whether different cell types within bone demonstrate differential expression patterns of clock genes in vivo. It would be of interest to further explore this using novel single‐cell sequencing‐based approaches,^(^ ^47^ ^)^ although cellular isolation of bone cells required for these techniques is challenging.^(^ ^48^ ^)^ Various genes involved in osteoclast activity (eg, *Ctsk*, *Nfatc*, *Rankl*, *Opg*) show potent diurnal expression patterns in bone,^(^ ^42^, ^43^, ^49^ ^)^ while the osteoblast markers *Runx2* and *Col1a1* were found not to be rhythmic.^(^ ^40^ ^)^ However, as *Runx2* is mostly involved in osteoblast differentiation^(^ ^50^ ^)^ and *Col1a1* lacks specificity for osteoblasts,^(^ ^51^ ^)^ this does not necessarily preclude rhythmic osteoblast activity. Genetic disruption of clock genes^(^ ^52^, ^53^, ^54^ ^)^ as well as environmental circadian disruption through shifting light/dark cycles^(^ ^40^ ^)^ have been shown to affect bone mass in mice, stressing the importance of circadian rhythm in bone remodeling for maintaining skeletal integrity.

### 2.1 Rhythmic bone resorption

Bone remodeling can be assessed in vivo by measuring markers of bone resorption and bone formation. Many clinical studies reported a diurnal rhythm in the bone resorption marker carboxy‐terminal collagen cross‐links (CTX), which is a product of bone collagen degradation. In healthy men and women, CTX levels in serum peak at night or early morning,^(^ ^55^, ^56^, ^57^, ^58^, ^59^ ^)^ indicating that bone resorption is particularly high during the resting phase. While mechanical unloading due to physical inactivity would be an obvious explanation for the increased bone resorption at night, CTX rhythm was found to be completely unaffected by timing of rest/activity.^(^ ^57^ ^)^ Variation in CTX is also not explained by diurnal variation in cortisol,^(^ ^57^, ^60^ ^)^ nor by light input,^(^ ^57^ ^)^ but CTX is known to be significantly reduced upon fasting,^(^ ^57^ ^)^ suggesting that rhythm in bone resorption is primarily mediated through cycles of fasting/feeding. This was confirmed in a randomized study in postmenopausal women showing that bone resorption is reduced upon food intake and increased upon prolonged fasting.^(^ ^61^ ^)^ These results could partly be reproduced by exogenous and endogenous insulin stimulation.^(^ ^61^ ^)^ The effect of food intake on bone remodeling is also mediated by gut‐derived incretin hormones, including glucose‐dependent insulinotropic polypeptide (GIP), glucagon‐like peptide 1 (GLP‐1), and glucagon‐like peptide 2 (GLP‐2). There are receptors for GIP and GLP‐1 present on osteoblasts, and receptors for GLP‐2 on osteoclasts, as nicely reviewed by Yavropoulou and colleagues.^(^ ^62^ ^)^ In line with this notion, oral glucose loading results in an acute and more prominent decrease in bone resorption than intravenous glucose loading.^(^ ^63^ ^)^ The response involves somatostatin, since the effect of oral glucose on bone can be abolished by octreotide.^(^ ^64^ ^)^ Of note, rhythmic bone resorption remains present during fasting, although less pronounced.^(^ ^65^ ^)^ Thus, fasting/feeding cycles likely have an effect on bone resorption independent of the SCN, but the exact underlying mechanisms remains uncharacterized.

It has been proposed that PTH also contributes to rhythmic activity in bone resorption. PTH levels demonstrate a diurnal rhythm, with peak values at night and a nadir in the morning.^(^ ^66^ ^)^ In turn, PTH rhythmicity may also be a reaction to rhythmic food intake, since PTH is the most important player in the negative feedback loop aiming for stable serum calcium levels within a narrow window. Serum PTH and calcium levels have a strong bidirectional and temporal relationship.^(^ ^67^ ^)^ Theoretically, an absence of dietary calcium intake at night will result in a subsequent increase in PTH. Indeed, PTH rhythmicity is diminished by prolonged fasting, at least in healthy premenopausal women.^(^ ^65^ ^)^ Fasting resulted in an increase in serum calcium levels and bone resorption, which could explain a subsequent decrease in PTH as a result from the negative feedback loop. Human subjects in which PTH rhythm was blunted by continuous calcium infusion still showed a diurnal pattern in urinary excretion of amino‐terminal collagen cross‐links (NTX),^(^ ^68^ ^)^ a bone resorption marker comparable to CTX. Intrinsic PTH rhythmicity may in itself thus contribute to rhythmic bone resorption, but this will be overruled in case of disturbances in the calcium homeostasis. Of note, long‐term PTH exposure should be discriminated from intermittent PTH peaks. It is well known that administration of daily intermittent doses of PTH in humans promotes bone formation and increases bone mass by stimulating osteoblasts without inducing the osteoclastogenesis that occurs with continuous exposure to PTH.^(^ ^22^, ^69^ ^)^ Therefore, we anticipate that short‐lived circadian peaks in PTH are more likely to affect osteoblast activity than osteoclast activity. Future studies are needed to identify the potential role of PTH and other calcium‐regulating hormones in rhythmic bone formation. For example, osteocalcin and vitamin D demonstrate a diurnal rhythm in humans^(^ ^70^, ^71^, ^72^ ^)^ and are known to modulate clock gene expression in bone of rats,^(^ ^73^, ^74^ ^)^ but they are mainly regulators of calcium homeostasis rather than major determinants of rhythmic osteoclast activity and bone resorption.

GCs directly affect the core clock in several ways, for example, by stimulation of *Per1/2* expression, to act as an extrinsic driving force on the intrinsic oscillators.^(^ ^75^ ^)^ Bone rhythms are GC sensitive: Rhythmic bone resorption depends on rhythmic GCs. Circadian oscillations in gene expression in osteoclasts are affected by GC signaling. The synthetic GC dexamethasone has been shown to induce rhythmic gene expression in cultured osteoclasts,^(^ ^42^, ^43^ ^)^ and GC depletion by adrenalectomy in mice abolishes rhythm in clock genes as well as osteoclast‐related genes in bone.^(^ ^42^ ^)^ A single injection of GCs can restore circadian gene expression in adrenalectomized mice,^(^ ^42^ ^)^ demonstrating the potency of GCs as a circadian timing signal for bone. Because GCs have been shown to stimulate the production of RANKL by osteoblasts, this thus promotes osteoclastic bone resorption through RANK‐RANKL signaling,^(^ ^76^ ^)^ which in itself has been shown to be rhythmic.^(^ ^77^ ^)^ However, GC‐dependent bone resorption is not rhythmic.^(^ ^57^, ^60^ ^)^ This is in contrast to osteocalcin for which rhythmicity depends on rhythmic cortisol expression^(^ ^60^ ^)^ as described below.

Although osteocytes have been suggested to mediate rhythmic bone resorption by promoting osteoclast differentiation and function,^(^ ^78^ ^)^ the osteocyte marker sclerostin does not show diurnal variation in human subjects, nor does it predict variation in CTX levels.^(^ ^55^ ^)^ Also, while global and osteoblast‐specific *Bmal1* deficiency in mice results in a low bone mass phenotype due to enhanced osteoclastogenesis and increased bone resorption,^(^ ^54^, ^77^ ^)^ this phenotype was not recapitulated by osteocyte‐specific *Bmal1* deletion.^(^ ^77^ ^)^

### 2.2 Rhythmic bone formation

Albeit less pronounced than markers of bone resorption, markers of bone formation also demonstrate 24‐hour serum profiles. One study found a minor rhythm in the commonly used bone formation marker procollagen type 1 N‐terminal propeptide (P1NP) across different ethnic groups,^(^ ^71^ ^)^ but this was not observed in many other studies.^(^ ^55^, ^58^, ^79^, ^80^ ^)^ The bone matrix protein osteocalcin, which is produced by active osteoblasts, shows a more robust diurnal rhythm in human serum.^(^ ^58^, ^59^, ^81^, ^82^ ^)^ As with CTX, osteocalcin levels are higher at nighttime compared with daytime, suggesting that osteoblast activity and therefore bone formation is also highest during the resting phase. Osteocalcin levels are not affected by fasting,^(^ ^65^ ^)^ but elimination of the morning peak in cortisol abolishes the expected morning decrease in osteocalcin in healthy individuals.^(^ ^60^, ^83^ ^)^ Thus, circadian GC rhythm is an important determinant of diurnal variation in osteoblast activity, which is supported by in vitro studies showing that dexamethasone can induce a rhythm in osteoblasts^(^ ^46^ ^)^ and that the endogenous GC peak has an inhibitory effect on bone formation. In fact, endogenous GCs are required to maintain bone health, as demonstrated by diminished osteoblast differentiation and progressive bone loss upon strongly attenuated GC signaling in mice through adrenalectomy^(^ ^84^ ^)^ and osteoblast‐specific disruption of GC action.^(^ ^85^, ^86^, ^87^ ^)^ However, excess GCs negatively impact bone formation by attenuating osteoblast differentiation.^(^ ^85^, ^88^ ^)^ GCs affect the Wnt signaling pathway, a critical regulator of osteoblastogenesis, in a dose‐dependent manner, with upregulation of Wnt at lower doses and downregulation at higher doses.^(^ ^89^ ^)^ It can be postulated that low GC concentrations observed at the natural trough of circadian GC rhythm could enable bone formation, while peak levels may have an inhibitory effect. However, this remains speculative and requires further investigation.

In contrast to GC signaling, which affects both osteoblasts and osteoclasts, signaling through adrenergic receptors modulates rhythm selectively in osteoblasts. The β‐adrenergic receptor agonist isoprenaline was found to promote clock gene oscillations in cultured osteoblasts^(^ ^45^, ^46^ ^)^ but not osteoclasts.^(^ ^42^ ^)^ Consistent with these observations, genetic ablation of α1‐adrenergic receptor signaling in mice disrupts the expression of osteoblast‐related and clock genes in bone,^(^ ^44^ ^)^ and treatment of mice with the α‐adrenergic receptor antagonist prazosin lowers bone mass by reducing bone formation.^(^ ^90^ ^)^ These findings collectively indicate that bone formation may be regulated through sympathetic nerve system activity, which is known to transmit signals from the SCN to peripheral tissue clocks. Nevertheless, additional studies are needed to confirm a direct relationship between adrenergic signaling, the circadian clock in bone, and rhythmic bone remodeling.

Bone formation is also regulated by melatonin, a hormone produced by the pineal gland in response to photic input from the SCN, at least in rodents. Melatonin release is inhibited by light and peaks during the dark phase when markers of bone formation are also high. Accordingly, melatonin has been found also to stimulate human osteoblast differentiation and proliferation in vitro^(^ ^91^, ^92^ ^)^ and to promote bone formation in animal models in vivo.^(^ ^92^, ^93^ ^)^ Melatonin depletion by pinealectomy as well as long‐term melatonin administration affect circadian oscillations in bone formation markers in rats,^(^ ^94^ ^)^ suggesting a role of melatonin in rhythmic bone formation. However, melatonin administration does not affect circulating osteocalcin levels or bone density in humans.^(^ ^95^ ^)^ In addition, melatonin has been shown to suppress activation of osteoclasts in mice through downregulation of RANKL on osteoblasts,^(^ ^96^ ^)^ but there is no significant relationship between melatonin rhythm and NTX rhythm in humans.^(^ ^97^ ^)^ These studies demonstrate that at least in humans, melatonin rhythm may not be an important determinant of bone remodeling.

### 2.3 Rhythmic bone growth

Like bone formation and bone resorption, it can be understood that skeletal growth is time‐of‐day dependent.^(^ ^98^ ^)^ Growth plates and their chondrocytes exhibit a strong circadian expression pattern of clock genes,^(^ ^99^ ^)^ including *Bmal1*.^(^ ^100^ ^)^ BMAL1 appears critical to growth plate development, as *Bmal1*‐deficient mice have significantly shorter femora and tibias.^(^ ^54^ ^)^ It is even suggested that a chondrocyte‐specific peripheral clock might exist,^(98^ ^)^ which could be an interesting topic for further research. PTH directly regulates circadian oscillation of the clock genes in chondrocytes^(^ ^101^ ^)^ and is able to reset the robust circadian rhythm in chondroprogenitor cells.^(^ ^102^ ^)^ This circadian oscillation is not only crucial for bone growth during aging but also important for endochondral bone formation during fracture healing.^(^ ^103^ ^)^ Besides, FGF23 expression, which itself depends on the circadian clock, food intake, and sympathetic activity^(^ ^104^ ^)^ and phosphate influence skeletal growth in a circadian fashion.^(^ ^105^ ^)^ The circadian rhythmicity within growth plates seems thus an interesting field for further research.

## 3 Chronotherapy of Osteoporosis

### 3.1 Prevention of osteoporosis associated with circadian disruption

Preventing disruption of the circadian timing system can be used to improve bone health of the general population. Shift work is likely the most widespread lifestyle associated with osteoporosis. Postmenopausal nurses who work in rotating shifts were reported to have a lower bone mineral density at the lumbar spine as well as femoral neck bones compared with daytime workers of the same age and sex.^(^ ^28^ ^)^ Although more than 25% of shift workers had osteoporosis at the lumbar spine (defined by a *T*‐score < −2.5), this was not observed for any of the daytime workers. In addition, women who worked in night shifts for more than 20 years demonstrated a significantly increased risk of wrist and hip fractures.^(^ ^27^ ^)^ These negative effects of shift work on bone could be a direct consequence of disruption of the circadian timing system. However, shift workers have also been suggested to maintain an overall unhealthier lifestyle (eg, increased smoking, a higher alcohol intake, lower physical activity, and altered eating habits), which may be a confounding factor. Moreover, shift workers and day workers may not be comparable with respect to their income and the type of work they perform. Nevertheless, a recent intervention study showed that circadian disruption in combination with sleep restriction also decreases P1NP levels in healthy subjects,^(^ ^106^ ^)^ pointing toward reduced bone formation. We have recently demonstrated that repeated shifts in light/dark cycle in mice, as a way to mimic human shift work, reduces markers of bone remodeling (ie, P1NP and CTX) and alters the material and structural properties of bone.^(^ ^40^ ^)^ In addition, continuous light exposure has been shown to diminish bone volume and density in mice.^(^ ^107^ ^)^ This could be the result of a disrupted circadian clock in combination with sleep deprivation, as sleep duration positively correlates to bone stiffness in humans,^(^ ^108^ ^)^ and short sleep is associated with low bone mineral density.^(^ ^109^, ^110^ ^)^ These studies collectively support a causal relationship between environmental circadian disruption and osteoporosis. Although it is unclear whether the increased risk of osteoporosis in shift workers is a consequence of excessive or inadequate bone turnover, circadian rhythms are markedly dampened in older individuals,^(^ ^111^ ^)^ indicating that osteoporosis associated with circadian disruption and aging could have a shared etiology.

Considering the large number of individuals with irregular working schedules (ie, nowadays more than 20% of the population in industrialized societies is involved in some form of shift work), strategies to preserve bone health in shift workers are highly warranted. Importantly, such strategies may also reduce the risk of age‐related osteoporosis in these individuals, by strengthening intrinsic circadian rhythms. Prevention‐focused strategies should preferably involve lifestyle interventions instead of pharmaceutical therapies that are costly and often accompanied by adverse effects. Two promising intervention strategies come to mind, ie, timed eating and timed exercise. In the previous sections, we discussed the importance of fasting/feeding cycles in regulating rhythmic bone remodeling. As bone resorption is increased by fasting at night and suppressed by feeding throughout the day, rhythmic patterns of food intake could strengthen rhythmic bone remodeling. Moreover, timing of food intake has been shown to entrain rhythm in a variety of peripheral tissues^(^ ^32^ ^)^ as well as in the circadian master clock (ie, the SCN),^(^ ^112^ ^)^ which was classically thought to be only affected by light. We have shown that time‐restricted feeding improves adaptation to shifting light/dark cycles in mice,^(^ ^49^ ^)^ indicating that timed eating could be an effective strategy to limit circadian disruption in shift workers.

In addition to food intake, physical activity has well‐characterized effects on bone metabolism. Mechanical forces that are exerted on bone through exercise, depending on their direction and magnitude, reduce the production of sclerostin by osteocytes.^(^ ^113^ ^)^ This relieves the inhibition of sclerostin on the Wnt signaling pathway, thereby promoting osteoblast differentiation.^(^ ^114^ ^)^ In addition, Wnt signaling promotes the expression of osteoprotegerin (OPG) by osteoblasts,^(^ ^115^ ^)^ which is a decoy receptor for RANKL, thus preventing RANK‐RANKL interaction and osteoclastogenesis. These dual effects of Wnt on osteoblasts and osteoclasts result in a net increase in bone mass.^(^ ^116^ ^)^ As such, high‐force exercise such as progressive resistance strength training mildly increases bone density in postmenopausal women^(^ ^117^ ^)^ and is proposed as a safe and effective way to prevent bone loss. Although it remains to be investigated whether timed exercise also entrains rhythm in bone, exercise during the night shift has been shown to adapt circadian temperature rhythms in humans,^(^ ^118^ ^)^ stressing the importance of physical activity as a circadian timing signal. Moreover, exercise may be used to adjust a person's late acrophase into early acrophase, as the former is associated with increased fall risk.^(^ ^119^ ^)^ Thus, both timed feeding and timed exercise may be implemented to prevent disruption of the biological clock and reduce the associated risk of osteoporosis.

### 3.2 Prevention of osteoporosis by correct timing of bone‐sparing agents

As bone metabolism exhibits potent circadian rhythms, it is not surprising that the response of bone to pharmacological treatment is time dependent. Various studies have shown that application of chronotherapy, ie, a method of treatment in which the administration of medication is coordinated with the biological clock, is an effective strategy to improve therapeutic efficacy of osteoporosis medication. Treatment of postmenopausal osteoporotic women with the recombinant PTH teriparatide differentially affects diurnal rhythm in bone turnover markers depending on the time of administration.^(^ ^80^ ^)^ While subjects treated with teriparatide in the evening showed a pronounced peak in serum CTX at night, morning teriparatide administration more effectively diminished CTX levels, indicating that timing of teriparatide treatment could be important. Accordingly, 12‐month treatment of osteoporotic women with teriparatide in the morning was shown to be more effective in increasing bone mineral density compared with treatment in the evening.^(^ ^120^ ^)^ Although as yet not clinically relevant, the effect of timed calcitonin treatment is illustrative: Healthy women who were treated with oral calcitonin just before dinner or in the evening showed a larger reduction in CTX compared with treatment in the morning,^(^ ^121^ ^)^ in line with the opposing actions of PTH and calcitonin. However, differences in baseline CTX levels and fasting state are confounding factors in this study. For many novel drugs to treat osteoporosis, chronotherapy is likely relevant yet unexplored. These drugs include abaloparatide, a PTH‐related protein that has recently been shown to have superior anabolic effects on bone compared with teriparatide,^(^ ^122^, ^123^ ^)^ and selective estrogen receptor modulators (SERMs) such as raloxifene^(^ ^124^, ^125^ ^)^ and bazedoxifene,^(^ ^126^, ^127^, ^128^ ^)^ which are used to prevent and treat postmenopausal osteoporosis. Chronotherapy may be less relevant for the current standard treatment with bisphosphonates because of their very long half‐life,^(^ ^129^ ^)^ although chronotherapy might improve the poor oral bioavailability (<1% of the administered dose)^(^ ^130^ ^)^ due to diurnal variation in intestinal absorption. In addition, monoclonal antibodies such as denosumab, which targets RANKL, and the more novel romosozumab, which targets sclerostin, have a half‐life of a few weeks,^(^ ^131^, ^132^ ^)^ rendering chronotherapy to increase therapeutic efficacy of these compounds less likely. Nevertheless, 39% of clinical trials of drugs with a long half‐life (more than 15 hours) still show dosing time dependence,^(^ ^133^ ^)^ indicating that variability in pharmacokinetics across the 24‐hour day could also modulate the effectiveness of longer‐acting drugs. Moreover, time‐of‐day‐dependent variation in pharmacokinetics is an important determinant of adverse drug effects, as explained below.

The bioavailability of a drug is dependent on circadian timing of the intestine, liver, and kidneys, which together importantly regulate the absorption, distribution, metabolism, and excretion of a drug.^(^ ^134^ ^)^ Accordingly, if biological timing is considered when administering a drug, this could not only optimize therapeutic outcomes but also reduce adverse effects. Indeed, a proof‐of‐principle study demonstrated that treatment of osteoporotic rats with vitamin D at the start of the dark phase instead of the light phase is not only more effective but also results into fewer side effects, such as hypercalcemia.^(^ ^135^ ^)^ This thus indicates that chronotherapy is a promising strategy to reduce or prevent adverse effects associated with osteoporosis treatment in humans, such as hypercalcemia‐induced dizziness and leg cramps in the case of PTH treatment.^(^ ^69^ ^)^

Another way in which chronotherapy could reduce negative effects on bone is by preventing GC‐induced osteoporosis, which is the most common cause of secondary osteoporosis.^(^ ^136^ ^)^ It was long thought that negative effects of GCs on bone are solely attributable to supraphysiological GC levels resulting from GC treatment, but we have recently shown that a disturbed GC rhythm could also contribute substantially.^(^ ^137^ ^)^ This finding is in line with the pivotal role of GC rhythm in mediating healthy bone remodeling, as discussed above, and emphasizes the importance of timing of GC therapy to limit its adverse effects.

## 4 Concluding remarks

**Fig 3:**
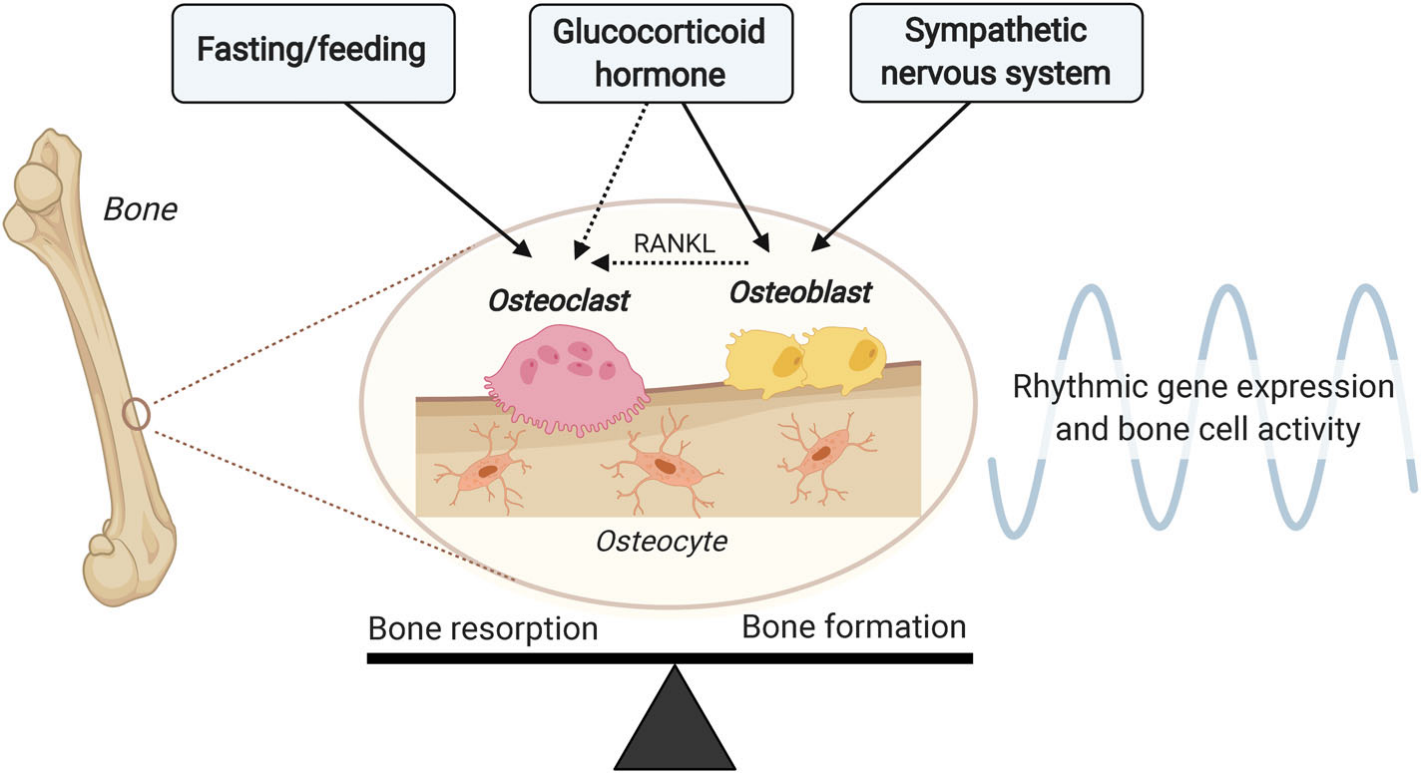
Circadian regulation of bone resorption by osteoclasts and bone formation by osteoblasts. Healthy bone depends on a delicate balance between bone resorption by osteoclasts and bone formation by osteoblasts. Studies indicate that these processes are strongly regulated by the circadian timing system, thereby producing diurnal variation in bone remodeling. While cycles of fasting/feeding primarily dictate osteoclast activity, sympathetic nervous system activity may selectively regulate rhythmic osteoblast activity. Glucocorticoid (GC) signaling is likely important for rhythm in both bone resorption and bone formation, as GCs can modulate the cellular clock of osteoclasts and osteoblasts, respectively. Of note, GCs could also affect rhythm in osteoclast activity indirectly through osteoblasts (as indicated by the dashed arrow), by regulating expression of the RANKL.

It has become clear that circadian rhythm plays a pivotal role in maintaining bone health by regulating bone remodeling. Evident rhythms in osteocyte activity have not (yet) been observed, but based on pronounced rhythms in the bone resorption marker CTX and the bone formation marker osteocalcin, combined with the data on rhythmic gene expression and the effects that hormones such as GCs have on these rhythms, we may conclude that both osteoclasts and osteoblasts possess robust diurnal activity patterns in humans with a peak during the rest phase. While rhythm in osteoclastic bone resorption is primarily regulated through fasting/feeding cycles and potentially fine‐tuned by GC hormone levels, rhythm in osteoblastic bone resorption seems mainly mediated by GC hormone levels and sympathetic nervous system activity, as illustrated in Fig. 3. Calcium‐regulating hormones and melatonin have also been implicated in rhythmic bone remodeling in cell cultures and rodents, but so far this is not supported by evidence in humans. Although a solid foundation has been laid, critical knowledge on the exact regulatory mechanisms of rhythmic bone remodeling is still scarce, and the importance of additional time cues, such as rhythm in physical activity and feeding, remains to be investigated. Future studies should also focus on investigating whether osteoclasts and osteoblasts are differentially regulated by different circadian timing signals and how this could be manipulated to promote bone strength and reduce bone remodeling disorders.

A significant part of the general population suffers from circadian disruption, thereby increasing the incidence of the bone remodeling disorder osteoporosis. Best established, osteoporosis risk is increased in the growing population of shift workers, which could be attenuated by promising novel interventions that prevent disruption of the circadian timing system, such as time‐restricted eating and/or time‐restricted exercise. In addition, osteoporosis risk may be increased in many individuals through mistimed administration of synthetic GCs, which disrupts the endogenous cortisol rhythm. Appropriate timing of GC medication could alleviate the risk of GC‐induced osteoporosis.

A critical knowledge gap remains for optimally timed anabolic or antiresorptive treatment: Too little is known yet to advise at which time medication should be administered, not only for optimization of therapeutic efficacy but also for prevention of side effects. Although chronotherapy is a very intuitive and promising strategy to improve therapeutic efficacy and reduce adverse effects, it is not yet broadly applied in clinical practice. A recent evaluation of clinical trials reported that less than 0.2% of currently ongoing trials involve a form of circadian intervention, and of those that do involve a form of chronotherapy, only 1% is focused on diseases of muscle, bone, and cartilage.^(^ ^138^ ^)^ These numbers emphasize the need for additional clinical studies that investigate circadian interventions to prevent and/or treat bone diseases such as osteoporosis. Thus, implementation of chronotherapy is crucial and should be further investigated, both for novel and existing therapies.

## AUTHOR CONTRIBUTIONS

**Elizabeth Winter:** Conceptualization; formal analysis; methodology; resources; ; validation; writing‐original draft; writing‐review & editing. **Sander Kooijman:** Writing‐review & editing. **Natasha Appelman‐Dijkstra:** Writing‐review & editing. **Onno Meijer:** Writing‐review & editing. **Patrick Rensen:** Writing‐review & editing. **Maaike Schilperoort:** Conceptualization; formal analysis; methodology; resources; validation; writing‐original draft; writing‐review & editing.
